# How traditional Chinese medicine can prevent recurrence of common bile duct stones after endoscopic retrograde cholangiopancreatography?

**DOI:** 10.3389/fphar.2024.1363071

**Published:** 2024-04-10

**Authors:** Haoyu Bian, Liping Zhang, Yupu Yao, Fuqi Lv, Jiaoyang Wei

**Affiliations:** ^1^ Department of Gastroenterology, Dongfang Hospital of Beijing University of Chinese Medicine, Beijing, China; ^2^ Graduate School, Beijing University of Chinese Medicine, Beijing, China

**Keywords:** cholelithiasis, common bile duct stones, recurrence, gut-liverbile acid axis, traditional Chinese medicine, pathogenesis, prevention

## Abstract

Common bile duct stones, as a type of cholelithiasis, are a benign biliary obstruction that easily acute abdominalgia, and Endoscopic Retrograde Cholangiopancreatography (ERCP) is usually the first choice for clinical treatment. However, the increasing recurrence rate of patients after treatment is troubling clinicians and patients. For the prevention of recurrence after ERCP, there is no guideline to provide a clear drug regimen, traditional Chinese medicine however has achieved some result in the treatment of liver-related diseases based on the “gut-liver-bile acid axis”. On the basis of this, this article discusses the possibility of traditional Chinese medicine to prevent common bile duct stones (CBDS) after ERCP, and we expect that this article will provide new ideas for the prevention of recurrence of CBDS and for the treatment of cholelithiasis-related diseases with traditional Chinese medicine in future clinical and scientific research.

## Introduction

Cholelithiasis is a disease in which stones occur in the biliary system, including the gallbladder or bile ducts. According to the site of development, it can be divided into gallbladder stones and intra- and extra-hepatic bile duct stones, of which those in the common bile duct can be subdivided into primary and secondary choledocholithiasis according to different sites of stone formation. In developed countries, gallstones affect 10%–15% of the adult population (Stinton and Shaffer, 2012), and 10%–15% of these patients have coexisting common bile duct stones (CBDS). In China, however, common bile duct stone is one of the major causes of death caused by benign biliary tract diseases, and primary CBDS have accounted for 50% of the total prevalence of cholelithiasis over the past 50 years (Minhu, 2019). A survey of 11,342 surgical cases of cholelithiasis by the Chinese Society of Surgery during year 1983–1985 showed that 52.8% had gallbladder stones, 11.0% secondary stones, 20.1% primary stones, and intrahepatic choledocholithiasis accounted for 16.1% of the cases. Ten years later, among 3,911 surgical patients with cholelithiasis, the incidence of gallbladder stones had risen relatively up to 79.9%, the incidence of primary CBDS dropped to 6.1%, and intrahepatic bile duct stones were 4.7% (Minhu, 2019). It can be seen that the composition ratio of different types of cholelithiasis has changed with the changes in people’s living habits and dietary structure, but the prevalence of CBDS in rural is still higher than in urban areas, and the reasons for its incidence are not yet fully understood. CBDS is easily combined with biliary pancreatitis, severe acute cholangitis, biliary liver abscess and other critical illnesses that threaten patients’ lives. Endoscopic Retrograde Cholangiopancreatography (ERCP) is currently the preferred treatment for CBDS. Despite its advantages of less pain and faster recovery. Endoscopic Biliary Sphincterotomy (EST) in ERCP will damage the function of Sphincter of Oddi (SO), which will lead to a partial loss of SO function, and then the duodenal bacteria intrude into the bile duct more easily, which is an important risk factor for recurrence of CBDS (Lee et al., 2022). One study kept track of patient’s conditions after ERCP for up to 54.4 months and found that 16.52% (57/345) of patients experienced stone recurrence after ERCP, with a median time to recurrence of 10.25 months (Li et al., 2018). In terms of prevention of recurrence of choledochal stones after ERCP, the 2019 Endoscopic management of CBDS guideline developed by ESGE explicitly states that Ursodeoxycholic Acid or other choleretic agents are not recommended as treatment or prevention method of stone recurrence (Manes et al., 2019). Therefore, there is still a large gap to be filled on how to reduce the high recurrence rate of CBDS after ERCP.

## Mechanisms of gallstone formation

### Formation of cholesterol stones

Normal bile in the human body consists of three main lipids including 4% cholesterol, 24% lecithin and 72% bile salts and water. The cholesterol molecule in bile is an extremely strong hydrophobic molecule, which generally is very difficult to dissolve in water, but under physiological conditions, when bile acids, phospholipids and cholesterol are maintained at a certain concentration, the cholesterol molecules in bile are able to be fully dissolved. In addition to this, alterations in other components of bile can also lead to stones. For example, when both cholesterol and pigment stones are oversaturated with cholesterol in the bile, patients with cholesterol stones are more likely to exhibit cholesterol crystals and fast crystallization under the influence of pronucleating proteins and apolipoprotein E genotype (van Erpecum et al., 2003). The mechanism of cholesterol stone formation is not yet fully understood, but there is no doubt that the presence of cholesterol supersaturation in the bile is a prerequisite for the formation of cholesterol stones. The emergence of cholesterol supersaturation is closely linked to the expression of genes for related transporter proteins in the human body responsible for the regulation of cholesterol, bile salts, and phospholipids in the bile, known as the sterol efflux transporters, ABCG5/8, the bile acid export pump, ABCB11, and the phospholipid flip-flop transporter, ABCB4(Wang et al., 2022). When there is a decrease in the gene expression of those proteins, which will make it easier to precipitate cholesterol crystals and produce stones. In liver cells, these functional genes are regulated by Liver X Receptor (LXR) and Farnesoid X Receptor (FXR) (Gadaleta et al., 2010; Van Erpecum, 2011; Liu et al., 2023).

### Formation of pigmented stones

Bile pigment stones can be categorized into brown pigment stones and black pigment stones, and the mechanisms of their formation are quite different, but the core mechanism of stones’ formation is closely related to bilirubin (Vítek and Carey, 2012). Black pigment stones are formed when the body produces too much bilirubin due to hemolysis or other reasons, and bilirubin can be secreted by the liver into the bile, and the excess bilirubin increases the risk of binding with calcium ions in the bile to form calcium bilirubinate, which leads to black pigment stones (Sun et al., 2023a). These stones are often formed in sterile gallbladders, and their etiology is associated with the UTG1A1 gene and the SLO1B1 gene, as well as with ABCG5/8, which control the excretion of bilirubin from hepatocytes (Van Erpecum, 2011).

Brown pigment stones are mostly formed in bile ducts, where calcium bilirubinate is the main component, and the causes of stones’ formation are related to biliary bacterial and parasitic infections (Stewart et al., 2006). When bacteria colonize the biliary system, they increase the amount of unconjugated bilirubin and free bile acids in the bile by producing β-glucuronidase and detergent-resistant phospholipase A1 enzymes, resulting in bile precipitation and the formation of bile duct stones (Maki, 1966: Vítek and Carey, 2012). Bacteria also give bile a lithogenic tendency by altering the PH of the bile. The proportion of bile acids in the bile composition of patients with pigmented stones is also altered compared to the healthy population, for example, the proportion of conjugated bile acids in the bile is reduced, the G:T ratio in conjugated bile acids is reduced, and the ratio of primary bile acids to secondary bile acids is elevated in these patients (Guan et al., 2023). Thus, changes in bile acid profile also play an important role in the formation of brown pigment stones.

### Altered biliary tract dynamics and the formation of gallstones

The human biliary system consists of bile canaliculi, intrahepatic ducts, hepatic ducts, choledochal ducts, common bile ducts, SO and gallbladder, and when there is an obstacle to the normal physiological activities of the biliary system, the increase in bile retention time will also increase the chance of the formation of gallstones. Alterations in the bile acid profile, metabolism-related disorders such as thyroid disease and hyperlipidemia can lead to alterations in the normal physiologic activity of the bile ducts. It has been found that patients with Hypothyroidism are more likely to develop CBDS due to impaired biliary emptying, while patients with Hyperthyroidism are at greater risk of developing stones due to increased cholesterol reabsorption and the presence of more cholesterol in the bile composition (Ravi et al., 2023). Studies have also found that dogs with hyperlipidemia are more likely to develop gallbladder and liver related diseases due to decreased gallbladder motility (Villm et al., 2023). The body regulates the biliary system through humoral hormones and other cytokines, including cholecystokinin (CCK), which stimulates gallbladder contraction and bile secretion, as well as GLP-2 and FGF19, which promote gallbladder relaxation (Choi et al., 2006; Yusta et al., 2017; Metry et al., 2023). The secretion of these hormones and signaling molecules is closely linked to bile acids in the gut (Hansen et al., 2020; Miedzybrodzka et al., 2021; Nerild et al., 2023). The diastole of the gallbladder ensures the normal excretion of bile salts, cholesterol and phospholipids from the liver cells in order to reduce the amount of cholesterol in the serum and tissues of the body; while the regular contraction of the gallbladder ensures the power of the bile flow in the biliary system and reduces the time of bile retention, thus decreasing the probability of gallstones occurrence. When the CCK-regulated gallbladder and intestine are dysfunctional, the excess cholesterol in the biliary system is not only more likely to precipitate, but also subject to the effect of intestinal reabsorption, the cholesterol that has already been metabolized out of the body’s circulation will re-enter the body and become a risk factor for the recurrence of gallstones (Jiao et al., 2022; Sun et al., 2022).

With advances in recent research, it has been found that Interstitial Cells of Cajal (ICCs) play an important role in maintaining the normal motility of the gallbladder (Huang et al., 2009; Fu et al., 2021). For example, in guinea pigs with cholesterol cholelithiasis, the density and ultrastructure of ICCs in the gallbladder are altered (Huang et al., 2021). Chronic inflammatory response of the gallbladder also leads to ultrastructural damage of the ICCs which in turn affects the normal physiological function of the gallbladder smooth muscle, resulting in abnormal gallbladder activity (Zhang et al., 2022; Ding et al., 2023).

## The cause of recurrence of CBDS

For the treatment of CBDS, regardless of the site and the nature of the stone, when the stone is obstructed in the bile duct, in addition to the traditional surgical treatment, ERCP, which is less invasive and quicker to recover, is now more often chosen to treat CBDS. However, the probability of recurrence of the CBDS after ERCP is 4%–24% (Chae et al., 2021). There are various reasons for the recurrence of CBDS, such as periampullary duodenal diverticulum, diameter of common bile duct, biological factors and different treatment modalities (Kim et al., 2021; Wu et al., 2021). In conclusion, the risk of CBDS recurrence after ERCP appears to be increased when patients have the above recurrence factors.

### Dysfunction of SO causes recurrence of CBDS

**FIGURE 1:**
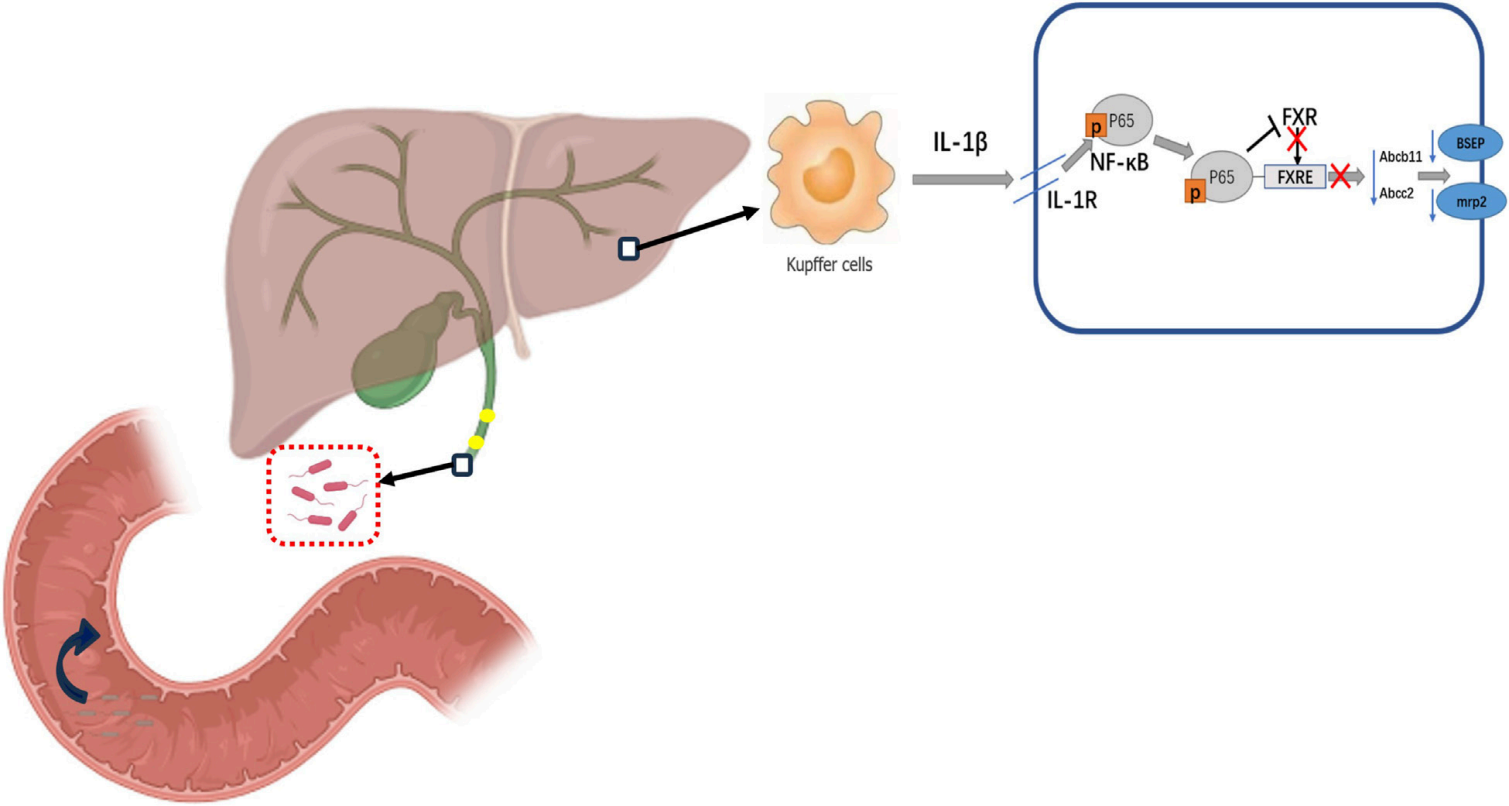
Dysfunction of SO causes recurrence of CBDS. After ERCP, duodenal bacteria entered the biliary system due to dysfunction of SO, which led to disruption of bile acid excretion in hepatocytes by affecting the NF-κB-mediated FXR signalling pathway in the liver.

While EST is effective in removing biliary stones, the procedure has also been shown to be a possible risk factor for recurrence of CBDS (Deng et al., 2019; Wu et al., 2021). Although some studies have also concluded that EST does not increase the recurrence rate of CBDS in 345 patients who underwent successful EST after at least 6 months (Li et al., 2018). The presence of biliary bacteria is inextricably linked to endoscopic maneuvers such as EST, in addition to the patient’s own SO dysfunction. Bacterial composition of the biliary system is more likely to be disturbed in patients with recurrent CBDS (Ye et al., 2020; Choe et al., 2021; Lee et al., 2022; Liu Q. et al., 2023b), and it has been found that the recurrence rate of patients after ERCP shows a gradual increase with the increase in the number of endoscopic lithotripsy procedures performed in patients (Kawaji et al., 2019; Wu et al., 2021), which suggests that there may be a possible association between high recurrence rates after ERCP and irreversible damage to the SO. Under normal physiological conditions, SO regulates the excretion of bile and pancreatic juice and prevents the reflux of duodenal contents. According to the findings of further research, dysfunction of the SO has been found to be an important risk factor for recurrence of CBDS. For example, relaxation of the SO can cause duodenal bacteria to enter the biliary tract, and exogenous β-Gase secreted by the bacteria can produce excessive unconjugated bilirubin, which in turn can lead to recurrence of CBDS (Zhang et al., 2020). Considering the flushing of bile and the inhibitory effect of bile salts, the human biliary environment should theoretically be a relatively sterile environment, but the dysfunction of the SO leads to the retrograde entry of duodenal bacteria into the biliary tract, which breaks the relative sterility of the biliary tract (Lyu et al., 2021). In conclusion, Dysfunction of the SO leads to a loss of function of the normal physiologic barrier of the biliary system, so it becomes easier for bacteria from the duodenal area to enter the biliary system by enterobiliary reflux. Chronic inflammation of the biliary system caused by enterobiliary reflux not only alters the relevant components of bile, but also activates the relevant inflammatory pathways affecting FXR in the liver, resulting in decreased expression of FXR-regulated bile salt export pump (BSEP) and multidrug resistance protein 2 (MRP2) and other relevant transport proteins (El Kasmi et al., 2018; Ghosh et al., 2021), Reduced expression of BSEP and MRP2 further affects bile composition and, together with other risk factors for recurrence that may be present in patients, the recurrence rate of CBDS remains high, As shown in Figure 1.

### Disturbance of the gut-liver-bile acids axis causes the recurrence of CBDS

**FIGURE 2:**
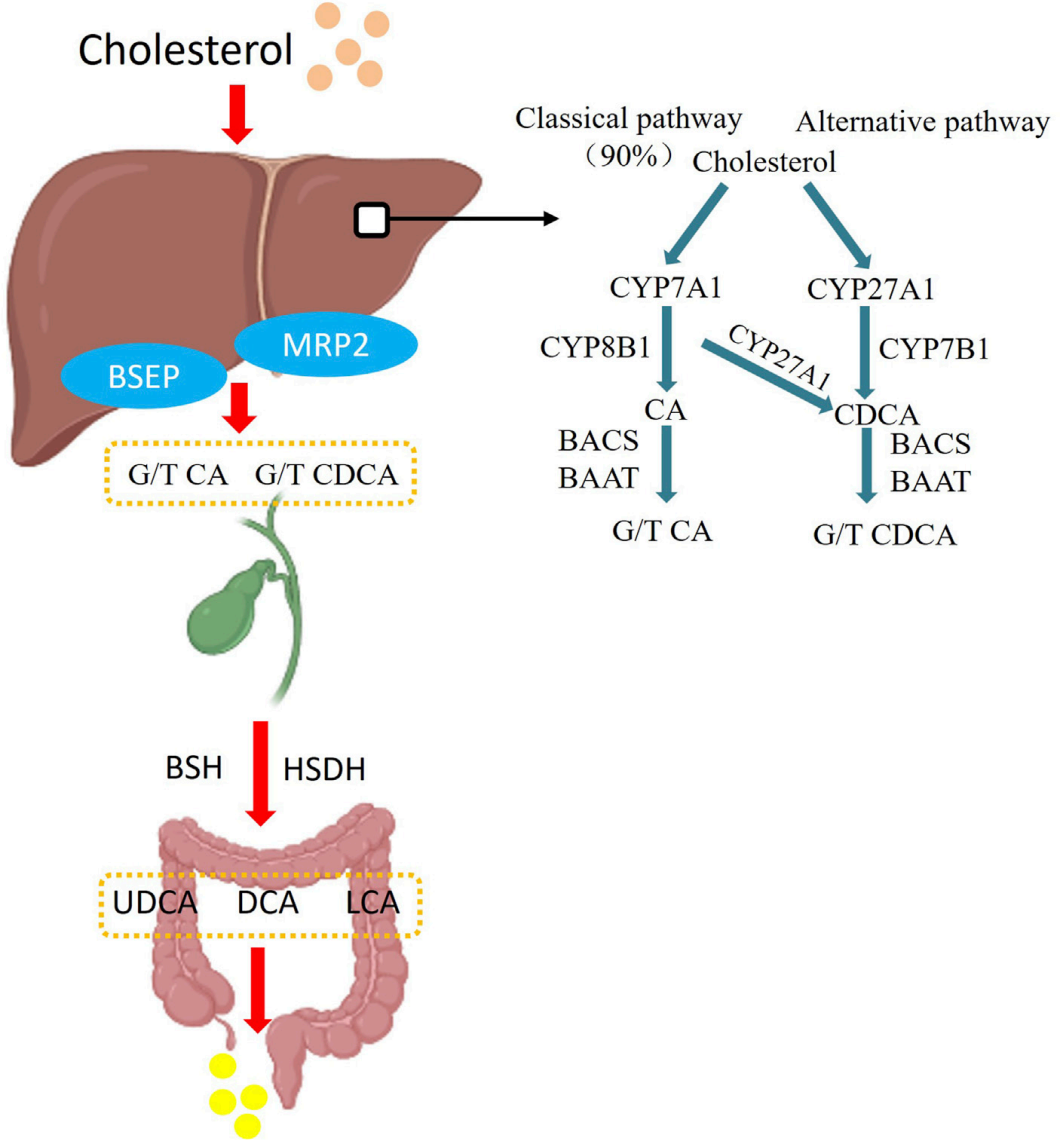
Bile acid synthesis pathway. In the classical synthetic pathway, Cytochrome P450 Family 7 Subfamily A Member 1 (CYP7A1) is the first step and the major rate-limiting enzyme in the classical pathway catalysing bile acid synthesis, followed by generation of cholic acid (CA) and chenodeoxycholic acid (CDCA) by Cytochrome P450 Family 8 Subfamily B Member 1 (CYP8B1) and Cytochrome P450 Family 27 Subfamily A Member 1 (CYP27A1) enzymes, with the classical pathway accounting for 90% of bile acid synthesis. In the alternative pathway, cholesterol is catalysed by CYP27A1 and CYP78B1 to produce CDCA-based bile acids, and the generated CA and CDCA are then modified by bile acid-CoA synthetase (BACS) and bile acid Coenzyme A: amino acid N-acyltransferase (BAAT), and then enter the intestinal tract along with the bile in the form of conjugated bile acids, and in the intestinal tract by bile salt hydrolase (BSH), Hydroxysteroid dehydrogenase (HSDH) to become deoxycholic acid (DCA), lithocholic acid (LCA), ursodeoxycholic acid (UDCA) and other secondary bile acids, and then eliminated from the body.

Bile acids have been shown to correlate with various types of biliary disorders, with significant decreases in the proportions of ursodeoxycholic acid (UDCA), chenodeoxycholic acid (CDCA), deoxycholic acid (DCA) and lithocholic acid (LCA) in the bile of patients with CBDS, patients with CBDS, the alteration of decreased G:T conjugation ratios in bile also resulted in increased hepatotoxicity of bile acid molecules (Guan et al., 2023). Combining taurine with bile acids also helps to promote the breakdown of lipids and bile acids for fat and weight loss. Disturbed microbiota in the biliary tract after ERCP not only lead to an increase in the proportion of hydrophobic bile acid molecules in the bile acid profile, which increases the risk of cholesterol molecules being precipitated, but also lead to abnormalities in the gut-liver-bile acids axis, which affects the normal physiological function of the liver to synthesize and excrete bile salts and lipids (Jetter and Kullak-Ublick, 2020). Under physiological conditions, bile acids are synthesized in liver particles, and cholesterol molecules are enzymatically linked through the classical pathway and alternative pathway to form cholic acid (CA) and CDCA, which are then enzymatically further conjugated to taurine or glycine to form T/G-CA and T/G-CDCA. The bile acids formed are passed through the bile and excreted into the digestive tract, where the primary bile acids are gradually formed into various secondary bile acids such as UDCA, DCA and LCA by the action of digestive tract bacteria (Ridlon et al., 2023), As shown in Figure 2. In the intestine, bile acid molecules are the most effective ligands for activating FXRs in intestinal cells, and bile acids are involved in the regulation of the organism’s gut-liver-bile acid axis by binding to FXRs. And there are differences in the binding capacity of different bile acid molecules to FXR. The binding capacity of bile acids is CDCA > DCA > LCA >> CA (de Aguiar et al., 2013).

Maeshall proposed the concept of gut-liver axis in 1998, which suggests that the liver and gut have not only anatomical homology, but also metabolic interactions and immunological correlations. Bile acids are an important link in the gut-liver axis. Bile acids molecules are synthesized in the liver and regulate the gut-liver axis by binding to the G protein-coupled bile acid receptor 5 (TRG5) and the FXR in the gut. In the gut, the microbiota, as a special class of being, can be indirectly involved in influencing the normal metabolic function of the liver by altering bile acids (Leung et al., 2016; Albillos et al., 2020; ðanić et al., 2018). When the biliary flora is disordered, overpopulated *Clostridium*, *Enterococcus*, Bifidobacterium, *Lactobacillus* and *Bacteroides* will decompose T/G-CA and T/G-CDCA into free bile acids by the effect of the bile salt hydrolase (BSH). These free bile acids are then progressively broken down into secondary bile acids by the effect of intestinal bacterial 7α-dehydroxylase (Chiang and Ferrell, 2019). Binding excess bile acids to FXR leads to excessive release of FGF19 from gut cells which in turn inhibits the bile acids synthesis pathway in the liver (Beuers et al., 2015), Further disorganization of the body’s bile acids pool increases the probability of CBDS recurrence.

## Traditional Chinese medicine to prevent recurrence of CBDS

Currently, the common treatments for CBDS include surgical treatment, endoscopic treatment, extracorporeal shockwave lithotripsy, laser lithotripsy, some chemical lithotripsy treatments that have been adopted more than 30 years ago (Helmstädter, 1999), and endoscopic dissolution of stones by drops of ethylenediaminetetraacetic acid into the nasobiliary tube (Lin et al., 1992). In addition, UDCA is commonly used in clinical practice for the oral treatment and prevention of stones, but UDCA is only effective for cholesterol stones in the treatment of cholelithiasis, and there is no clear medicinal treatment plan for the prevention of CBDS after ERCP. In traditional Chinese medicine theory, the occurrence of gallstones is closely related to abnormal liver function. Traditional Chinese medicine is a type of drug with multiple target effects, guided by traditional Chinese medicine theory and mainly based on plant-based drugs. In the prevention and treatment of gallstones, traditional Chinese medicine mainly restores the liver’s drainage function to regulate the composition and excretion of bile. With the clarification of the mechanism of traditional Chinese medicine in the treatment of various liver diseases, traditional Chinese medicine has gradually emerged in the prevention of CBDS after ERCP. In Table 1, we summarized literature on how traditional Chinese medicine can prevent the recurrence of CBDS.

**TABLE 1:** Traditional Chinese medicine prevent the recurrence of CBDS.

Traditional Chinese medicine	Major composition of traditional Chinese medicine	Research objects	Primary outcome measure	References
Danning tablets (DNts)	Extracts of Radix et Rhizoma Rhei, Rhizoma et Radix Polygoni Cuspidati, Pericarpium Citri Reticulatae, Pericarpium Citri Reticulatae Viride, Radix Curcumae, Fructus Crataegi and Rhizoma Imperatae	Rats with acute liver injury and cholestasis	Reducing oxidative stress and inflammatory infiltration in liver tissue	Ding et al. (2012)
		Asymptomatic T2MD patients after cholecystectomy	Improving glucose and lipid metabolism and increased the level of glucagon-like peptide-1	Chen et al. (2021)
		Mice with obesity-induced metabolic associated fatty liver disease (MAFLD) and related metabolic disorders	Down-regulating the hepatic protein levels of and its downstream factors	Ma et al. (2021)
		Rats with cholestasis	enhancing the expressions of hepatic canalicular efflux transporters (Bsep and Mdr2), renal efflux transporter Ostβ and bile acid-detoxifying enzyme (Cyp2b1 and Ugt1a1) and attenuate the hepatic Mrp2 translocation	Ding et al. (2014)
Yinchen decoction (TCD)	Artemisia capillaris Thunb	Mice with cholestatic liver injury	Activating the liver FXR/SHP and ileal FXR/FGF15 signaling pathways	Song et al. (2023)
Yinchenzhufu decoction	Artemisia capillaris Thunb, Atractylodes macrocephala Koidz, Zingiber officinale Rosc, Aconitum carmichaelii Debx, Glycyrrhiza uralensis Fisch and Cinnamomum cassia Presl	Mice with chronic cholestatic	Promoting the excretion and metabolism of BAs and inhibiting inflammation via the TLR4/NF-κB signaling pathway	Li et al. (2024)
Da-Chai-Hu decoction	Bupleuri Radix, Scutellariae Radix, Paeoniae Radix Alba, Pinelliae Rhizoma, Aurantii Fructus Immaturus, RheiRadixetRhizome, Zingiberis Rhizoma Recens and Jujubae Fructus	Mice with cholestatic liver injury	Activating the FXR signaling pathway to regulate the synthesis and excretion of BAs	Zhou et al. (2022)
Inchin-ko-to	Genipin	Normal rats	Stimulating the multidrug resistance-associated protein 2	Shoda et al. (2004)
Bupleuri Radix	Saikosaponins	Normal mice	Regulating FXR-related genes and transporters in the liver and intestine	Shoda et al., 2004; Wang et al., 2023
Astragali Radix	Total Astragalus saponins	Rats with Cholestatic Liver Fibrosis	Repressing liver inflammation, improving expressions of FXR-related pathway	Zhang et al. (2024)
DNts	Curcumin	Patients and mice with cholestatic liver	Regulating the FXR signaling pathway	Yang et al. (2016)
Gardeniae Fructus	Gardenia extract	Rats with intrahepatic cholestasis	Regulating bile acid enterohepatc circulation	Qin et al. (2024)
Pueraria lobata	Ethanol extract of Pueraria lobata	Rats with acute myocardial infarction	Regulating gut microbiota and bile acid metabolism	Zhang et al. (2023a)
Aconite	Aconite aqueous extract	Mouse model of diarrhea	Regulating gut microbiota and bile acid metabolism	Zhang et al. (2023b)
Coptis chinensis	Berberine	Mice with ulcerative colitis	Modulating the fecal-bacteria-related bile acid metabolism	Sun et al. (2023b)
Atractylodes lancea	Atractylodes lancea extracts	Mice with intestinal dysbacteriosis	Regulating the disorder of intestinal flora	Zhang et al. (2023c)
Chaihu-Shugan-San	Bupleurum chinense DC., Citrus reticulata Blanco, Cyperus rotundus L., Citrus × aurantium L., Ligusticum striatum DC., Paeonia lactiflora Pall., Glycyrrhiza uralensis Fisch	Mice with depression-like behavior	Altering the gut microbiota and levels of the bile acids hyocholic acid and 7-ketoDCA	Ma et al. (2022)
Xiayuxue decoction	Rheum officinale Baill., Prunus persica (L.) Batsch and Eupolyphaga sinensis Walker	Nude mice with hepatocellular carcinoma	Promoting primary bile acid synthesis and improving gut microbiota	Deng et al. (2024)
Rhubarb decoction	Rhubarb and dark plum	Rats with Minimal hepatic encephalopathy	Altering the gut microbiota	Du et al. (2023)
Qingre Lidan decoction	Yinchenhao, Xiakucao, Huangqin and Jinqiancao, Dahuang and Houpo	Rats with cholestatic liver injury	Regulating endogenous metabolites and microbiota disorders	Chang et al. (2023)
Yinchenhao Decoction	Geniposide and chlorogenic acid	Mice with non-alcoholic steatohepatitis	Improving the gut microbiome and activating FXR signaling pathway	Li et al. (2023)
Qiwei Baizhu Powder	Atractylodis Macrocephalae Rhizoma, Ginseng Radix et Rhizoma, Poria, Puerariae Lobatae Radix, Aucklandiae Radix, Pogostemonis Herba, and Glycyrrhizae Radix et Rhizoma	Mice with antibiotic-associated diarrhea	Regulating gut microbiota and bile acids	Xie et al. (2022)

### Traditional Chinese medicine prevents CBDS recurrence by regulating bile acid metabolism

In preventing the recurrence of CBDS, bile acid molecules not only directly participate in the formation of micelles in the bile to fully dissolve the stones, but also give the special molecular structure of bile acid a cleansing agent-like effect, which effectively removes excessive fats in the digestive system to prevent the formation of stones brought about by hyperlipidemia (Lefebvre et al., 2009). Bile acid molecules can also act as signaling molecules such as hyodeoxycholic acid (HDCA), which can prevent the formation of stones by inhibiting FXR in the mouse ileum, enhancing bile acid synthesis to reduce total cholesterol levels in liver, serum, and bile (Kuang et al., 2023; Shen et al., 2023; Zhong et al., 2023).

**FIGURE 3:**
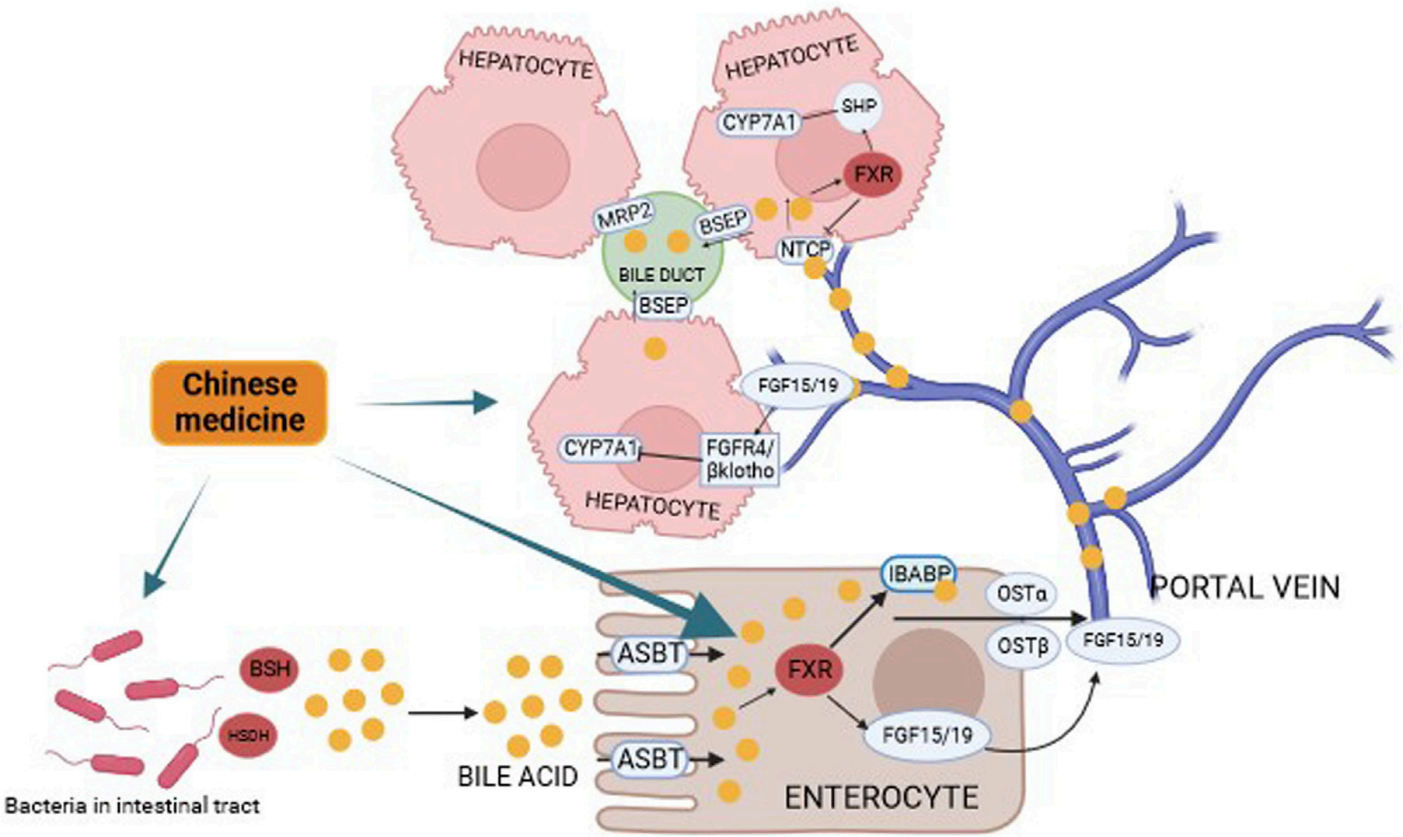
Prevention of CBDS recurrence by regulating the “gut-liver-bile acid” axis in Chinese medicine. Bile acids subjected to bacterial BSH and HSDH are reabsorbed by intestinal cells, and upon binding to FXR in hepatic cells, they affect the classical pathway in the bile acid synthesis pathway by activating the heterodimeric chaperone SHP, which in turn blocks the normal transcription of the CYP7A1 enzyme. Binding to FXR also reduces the concentration of effective bile acids by releasing FGF15/19 to form a heterodimeric complex with FGFR and β-Klotho, which in turn affects the normal transcription of CYP7A1 leading to an abnormality in the pathway of bile acid synthesis.

Recent studies have found that traditional Chinese medicine can prevent the recurrence of CBDS by regulating the expression of key enzymes in the bile acid synthesis pathway, regulating the expression of the FXR gene in the liver cells, and regulating the proteins responsible for the transport of bile acid molecules in the liver cells, thus regulating the homeostasis of the bile acid pool of the organism, in order to maintain the normal physiological function of the liver, and to achieve the prevention of CBDS. Since ancient China, the use of animal bile as medicine has been customary, and bear bile powder is an important traditional Chinese medicine with the effect of clearing heat and calming the liver. However, due to the fact that bear bile powder is not easy to obtain, it has been found that tauroursodeoxycholic acid (TUDCA) is the most important ingredient in bear bile powder by chemical means, and that the administration of TUDCA to Hyperlipidemic mice can significantly regulate the content of TC, TG, HDL-C and LDL-C in serum, and reduce the formation of cholesterol stone (Cui et al., 2023). In China, Danning tablets (DNts) are made from extracts of Radix et Rhizoma Rhei, Rhizoma et Radix Polygoni Cuspidati, Pericarpium Citri Reticulatae, Pericarpium Citri Reticulatae Viride, Radix Curcumae, Fructus Crataegi and Rhizoma Imperatae, which have proven to be effective in the treatment of liver and gallbladder diseases. DNts can protect liver tissue by reducing oxidative stress and inflammatory infiltration in liver tissue (Ding et al., 2012). And DNts can also maintain normal glucose-lipid metabolism in the body by activating FXR in the liver and intestinal tissues (Chen et al., 2021), In addition, DNts have also been shown to reduce the risk of gallstones by modulating the SREBP pathway to reduce various types of lipids in the body (Ma et al., 2021). In patients with CBDS altered hepatic physiology and downregulation of the corresponding transporter proteins often lead to cholestasis, and DNts intervention in a rat of cholestasis resulted in a significant upregulation of the expression of BSEP, MRP2 as well as renal Organic Solute Transporter Beta (Ostβ) in liver tissues of the animals (Ding et al., 2014). It shows that DNts can enhance hepatic bile excretion by regulating the corresponding bile acids transporter proteins in the liver, which on the one hand can reduce the disruption of normal metabolic functions in the liver due to cholestasis, and on the other hand, the cleansing and sterilizing ability of bile salts on the biliary system can reduce the overproliferation of microorganisms in the bile ducts. In addition to DNts, other traditional Chinese medicine compounds have also been shown to have a role in regulating hepatic metabolism. Yinchen decoction (YCD) has been used for the treatment of jaundice in China for nearly 1,000 years, and after 10 days of treatment with YCD in mice with intrahepatic cholestasis, it was demonstrated that YCD could restore the stability of the bile acid pool by regulating FXR/FGF15 (Song et al., 2023). Yinchenzhufu decoction, a classical formula, has also been shown to promote the excretion and metabolism of BAs and inhibiting inflammation via the TLR4/NF-κB signaling pathway (Li et al., 2024). Da-Chai-Hu decoction also stabilizes bile acid profiles (Zhou et al., 2022). And nowadays, more and more traditional Chinese medicine such as Inchin-ko-to or certain components of traditional Chinese medicine such as Saikosaponins, Total Astragalus saponins, Curcumin, Gardenia extract have been shown to have better efficacy in regulating FXRs, bile acid receptors, and in improving the pathway of bile acid synthesis in order to maintain the homeostatic state of bile acids in the body (Shoda et al., 2004; Yang et al., 2016; Wang et al., 2023; Qin et al., 2024; Zhang et al., 2024). As shown in Figure 3.

### Traditional Chinese medicine prevents CBDS recurrence by regulating the gut microbiota

The human intestinal tract is home to about 100 trillion bacteria, which, in addition to facilitating digestion and absorption, also play an important part in regulating the body’s internal environment. For example, in the intestines of patients with diarrhea-predominant irritable bowel syndrome (IBS-D), the proportions of DCA, 7-ketodeoxycholic acid, and lithocholic acid are elevated, which corresponds to alterations in functional genes controlling BA metabolism in the gut microbiota (Du et al., 2023). *Clostridium* sp. and increased secondary BAs, especially LCA and its derivatives (e.g., TLCA), occur in the gut of patients with Nonalcoholic Fatty Liver Disease (NAFLD), and these can cause disruption of the body’s internal environment (Zhang P. et al., 2023a). The relationship between gut microbiota and bile acids is considered to be a reciprocal one. It has been found that changes in the composition of intestinal flora in patients with NAFLD, Cholestatic liver diseases, affect the composition of the bile acids pool through the action of related enzymes, which in turn cause pathological changes in the liver and other tissues, and bile acids molecules, as a class of signaling molecules, can regulate the immune response by regulating the body’s immune response, which can lead to pathological changes in the liver. In addition, bile acids molecules can also regulate the composition of the gut microbiota by modulating the immune response (Winston and Theriot, 2020; Shao et al., 2021). It is worth mentioning that the microbiota in the organism has a role in the regulation of bile acids. Xin Ye and Dan Huang found that after 8 weeks of lithogenic-diet feeding, mice’ conditions were significantly improved in terms of gallstones, hepatic steatosis, and hyperlipidemia after the intervention of Limosilactobacillus reuteri strain CGMCC 17942 and Lactiplantibacillus plantarum strain CGMCC 14407. It is hypothesized that L. reuteri and L. planta rum treatments reduced the concentrations of T-a-MCA and T-b-MCA modulated FXR receptors in the gut, which in turn contributed to the treatment and prevention of CBDS (Ye et al., 2022).

Traditional Chinese medicine compounds such as Pueraria lobata, Aconite, Berberine, Atractylodes lancea and the traditional Chinese medicine compounds compound Chaihu-Shugan-San, Xiayuxue decoction have been shown to be effective in treating diarrhea, inflammatory bowel disease, depression and myocardial infarction by regulating the gut microbiota and improving the bile acids pool of the body in animal experiments (Ma et al., 2022; Sun et al., 2023a; Zhang et al., 2023b; Zhang et al., 2023c; Zhang et al., 2023d; Deng et al., 2024). In the recurrence of CBDS, the disorder of bile acids pool of the organism and the decrease of effective bile acids molecules are the risk factors, and traditional Chinese medicines have been proved to be effective in regulating the synthesis and metabolism of bile acids and improving the gut microbiota to regulate the gut-liver axis for the treatment of liver-related diseases, such as Rhubarb decoction, which can regulate the gut microbiota by enema, and can improve the bile acids profiles for the improvement of the liver function in rats (Du Y et al., 2023). Qingre Lidan decoction can regulate the body’s bile acids metabolism, inflammatory response and gut microbiota disorders, in order to alleviate the abnormalities of the body’s bile acids pool caused by inflammation of the gallbladder or biliary tract (Chang et al., 2023). Geniposide and chlorogenic acid, the main ingredients in Yinchenhao Decoction, also have significant effects in regulating the gut microbiota and thus affecting the FXR in order to alleviate Nonalcoholic Steatohepatitis (NASH) through the gut-liver axis (Li et al., 2023). Qiwei Baizhu Powder has similar effects (Xie et al., 2022). As shown in Figure 3.

### Traditional Chinese medicine prevents CBDS recurrence by regulating biliary tract dynamics

**FIGURE 4:**
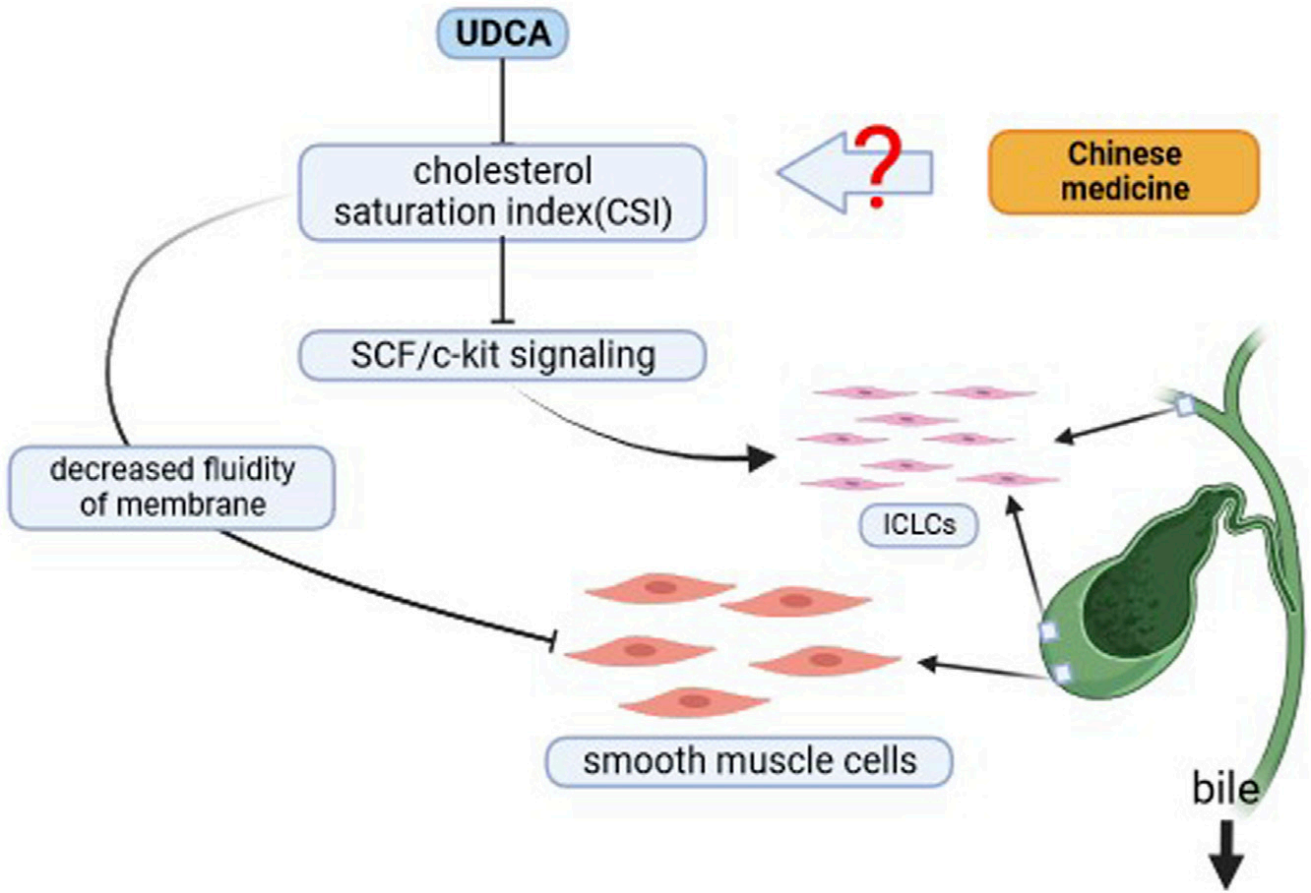
Chinese medicine strengthens the flow of bile. Can Chinese medicine affect the SCF/c-kit/ICLCs pathway and enhance bile flow by modulating cholestrol saturation index (CSI) and augmenting biliary smooth muscle cells.?

Gallbladder dysmotility resulting in slow bile flow is one of the most important factors for the production of gallstones in the biliary system, however, cholecystitis is the dominant cause of gallbladder dysmotility, when cholecystitis is present due to gallstones causes, the inflammatory response affects the gallbladder interstitial Cajal-like cells (ICLCs), leading to gallbladder dysmotility (Lin et al., 2019). It has been found that the higher the degree of cholesterol saturation in bile, the lower the density of ICLCs, which also suggests that the probability of gallbladder dysmotility is relatively higher in populations in which bile exhibits a tendency to lithogenicity (Pasternak et al., 2013). Among the many gastrointestinal hormones Cholecystokinin (CCK) has a role in stimulating gallbladder contraction, and CCK can modulate Common Bile Duct (CBD) contraction by stimulating CCK-A receptors on ICLCs(Xu et al., 2023). Telocytes (TCs), a new type of interstitial cells, have recently been identified in many organs, including gallbladder. Such mesenchymal stromal cells may be involved in CBD motility by engaging in signaling. Increases in lithogenic indexes such as the cholestrol saturation index (CSI) cause a decrease in the density of TCs, which in turn leads to gallbladder dysmotility (Matyja et al., 2013). UDCA is commonly used in the treatment of cholesterol stones as it is effective in increasing the degree of cholesterol solubilization by bile, i.e., CSI, and one study found that after 4 weeks of treatment with UDCA, the protective effect of UDCA on ICLCs in gallbladder tissues may be achieved through modulation of the TNF-alpha/Caspase8/caspase3 inflammatory pathway (Wan et al., 2018). This also inspired the question of whether traditional Chinese medicine could prevent the recurrence of CBDS by decreasing this inflammatory pathway and by lowering the body’s cholesterol level, which in turn regulates the function of ICLCs and enhances the contractility of the biliary system. Moreover, by effectively lowering the cholesterol level and normalizing the bile composition, the density of TCs in the biliary system can also be normalized, which in turn brings down the occurrence of stones (Pasternak et al., 2017). These studies also suggest the great potential of TCM in the treatment of such diseases. As shown in Figure 4.

## Concluding

In this article, we summarize the advantages of traditional Chinese medicine in the treatment of hepatobiliary related diseases. From various academic and clinical perspectives, such as gut-liver-bile acids axis and biliary tract dynamics. At the end of this article, we propose the possibility of TCM in preventing the recurrence of CBDS after ERCP. Hope this article can bring some different ideas to the scholars.

## Figures and Tables

**Table:** 

**ABCB4**	ATP-binding cassette, subfamily B member 4
**ABCB11**	ATP-binding cassette, subfamily B member 11
**ABCG5/8**	ATP-binding cassette, subfamily B member 5/8
**BAAT**	bile acid coenzyme A: amino acid N-acyltransferase
**BACS**	bile acid-CoA synthetase
**BSEP**	bile salt export pump
**BSH**	bile salt hydrolase
**CA**	cholic acid
**CBD**	common bile duct
**CBDS**	common bile duct stones
**CCK**	cholecystokinin
**CDCA**	chenodeoxycholic acid
**CSI**	cholesterol saturation index
**CYP7A1**	cytochrome p450 family 7 subfamily A member 1
**CYP27A1**	cytochrome p450 family 27 subfamily A member 1
**CYP8B1**	cytochrome p450 family 8 subfamily B member 1
**DCA**	deoxycholic acid
**DNts**	danning tablets
**ERCP**	endoscopic retrograde cholangiopancreatography
**ESGE**	European society of gastrointestinal endoscopy
**EST**	endoscopic biliary sphincterotomy
**FGF19**	fibroblast growth factor 19
**FXR**	farnesoid X receptor
**GLP-2**	glucagon-like peptide 2
**HDCA**	hyodeoxycholic Acid
**HDL-C**	high-density lipoprotein cholesterol
**HSDH**	hydroxysteroid dehydrogenase
**IBS-D**	diarrhea-predominant irritable bowel syndrome
**ICCs**	interstitial cells of cajal
**ICLCs**	interstitial cajal-like cells
**LCA**	lithocholic acid
**LDL-C**	low-density lipoprotein cholesterol
**LXR**	liver X receptor
**MCA**	muricholic acid
**MRP2**	multidrug resistance protein 2
**NAFLD**	non-alcoholic fatty liver disease
**NASH**	non-alcoholic steatohepatitis
**Ostβ**	organic solute transporter beta
**PH**	potential of hydrogen
**SHP**	small heterodimer partner
**SLO1B1**	solute carrier organic anion transporter family, member 1B1
**SO**	sphincter of oddi
**SREBP**	sterol-regulatory element binding proteins
**TCD**	yinchen decoction
**TCs**	telocytes
**TC**	total cholesterol
**TG**	triglyceride
**TRG5**	G protein-coupled bile acid receptor 5
**TUDCA**	tauroursodeoxycholic acid
**UDCA**	ursodeoxycholic acid
**UTG1A1**	recombinant UDP glucuronosyltransferase 1 family, polypeptide *A1*
